# Advances in metabolic flux analysis toward genome-scale profiling of higher organisms

**DOI:** 10.1042/BSR20170224

**Published:** 2018-11-23

**Authors:** Georg Basler, Alisdair R. Fernie, Zoran Nikoloski

**Affiliations:** 1 Bioinformatics Group, Institute of Biochemistry and Biology, University of Potsdam, Karl-Liebknecht-Str. 24-25, 14476 Potsdam-Golm, Germany; 2 Systems Biology and Mathematical Modeling Group, Max Planck Institute of Molecular Plant Physiology, Am Mühlenberg 1, 14476 Potsdam-Golm, Germany; 3 Central Metabolism Group, Max Planck Institute of Molecular Plant Physiology

**Keywords:** Constraint-based approaches, Genome-scale networks, Metabolic Flux Analysis, Metabolic Modeling, Metabolomics

## Abstract

Methodological and technological advances have recently paved the way for metabolic flux profiling in higher organisms, like plants. However, in comparison with omics technologies, flux profiling has yet to provide comprehensive differential flux maps at a genome-scale and in different cell types, tissues, and organs. Here we highlight the recent advances in technologies to gather metabolic labeling patterns and flux profiling approaches. We provide an opinion of how recent local flux profiling approaches can be used in conjunction with the constraint-based modeling framework to arrive at genome-scale flux maps. In addition, we point at approaches which use metabolomics data without introduction of label to predict either non-steady state fluxes in a time-series experiment or flux changes in different experimental scenarios. The combination of these developments allows an experimentally feasible approach for flux-based large-scale systems biology studies.

## Introduction

The last two decades of systems biology research have witnessed advances in high-throughput profiling technologies which allow for monitoring multiple cellular components, from DNA modifications [1], transcripts [2], small RNAs [3], to proteins, their posttranslational modifications [4], and metabolites [5]. The resulting data read-outs provide an unprecedented possibility to glean the functionality of different cellular layers, from gene regulation to protein–protein interactions and metabolism [6]. However, these proxies for abundance of molecules often do not directly provide information about the activity of the underlying biochemical reactions which affect the molecular pools. More specifically, differential behavior of the abundances in two different experimental scenarios does not necessarily imply that the associated reactions exhibit difference in activities, which can lead to erroneous conclusions about the functionality of biological systems.

Here, we focus on reviewing approaches for estimating the activity of metabolic reactions. Metabolism incorporates the entirety of biochemical reactions through which small molecules are interchanged with the environment and further processed to achieve a variety of cellular tasks, from development and growth to reproduction [7]. The approach for flux estimation is based on the idea of tracing the incorporation of labeled atoms from labeled substrate(s) into the molecules in a studied biological system [8]. The activities of intracellular metabolic reactions cannot be measured, but are estimated from data about labeling of molecular pools and a model specifying the relevant reactions which influence the molecular pools of interest. The resulting reaction rates (or fluxes), quantifying the reactions’ activities, are the integrated outcome of upstream processes, including (post)transcriptional and (post)translational, and the current metabolic state of the system. Hence, metabolic flux profiling at a genome-scale will allow a new level of comparative analyses closest to the functionality of a studied system [9]. Knowledge of activity of metabolic reactions also offers context-specific insights into the integration of various cellular processes with the environment, as it allows us to pinpoint which reactions and where in the system they are active. It also provides a reference state which one can use in the design of manipulation strategies for various biotechnological applications [10,11].

The abovementioned high-throughput profiling technologies have already resulted in genome-wide cell-type, tissue-, and organ-specific data atlases documenting the abundance of the various molecular components at a systems’ level. The cellular complexity of a biological system is tantamount to the challenges faced by genome-wide metabolic flux profiling. The principal reasons include (i) experiment design to ensure that the basic assumptions of the computational approaches for flux estimation are met, (ii) technological developments to facilitate measuring a sufficient number of (sub)cellular abundances, and (iii) computational issues posed by the integration of the resulting data.

To detail these reasons, in the following, we provide a succinct overview of the recent technological developments to gather data as well as the existing approaches for metabolic flux estimation, with a brief emphasis of their advantages and shortcomings. The aim of the review is to point at recent developments of metabolic flux profiling in both prokaryotes and eukaryotes which could pave the way for flux profiling at a genome-scale level in multicellular organisms, with focus on plants. We refer the reader to comprehensive reviews of flux profiling approaches [12–14] and their applications in microbial systems and plants [9,15].

## Advances in technologies to gather data for metabolic flux profiling

From an experimental viewpoint, there have been relatively few advances in recent years, with gradual shift away from using radioisotopes toward stable isotopes [16]. Given the fact that companies specializing in radiochemicals have dramatically reduced the number of labeled metabolites they offer, this shift is likely to rapidly gather pace. Stable isotope labeling experiments are generally evaluated by means of coupled mass spectrometry (MS) or nuclear magnetic resonance (NMR) spectroscopy with a wide range of methods being developed which allow very good coverage of primary metabolism. Approaches based on MS and NMR are fundamentally different with only the latter able to readily provide positional information [17], advantageous for flux estimation [18]. In addition, NMR can be used to monitor metabolism within living plant cells, which has proven informative in analyzing unidirectional reaction rates in response to changes in temperature or oxygen concentrations [19]. These advantages, however, come at a cost since NMR spectroscopy is considerably less sensitive than MS-based technologies.

The sensitivity of MS is its major advantage for flux estimation, but only limited positional information is readily available in a form of known fragmentation patterns of the metabolites [20–22]. The adoption of MS-based technologies has expanded flux profiling studies beyond the core energy metabolism allowing flux estimation for amino acid, phenylpropanoid, terpene, and benzoid metabolism [23–25].

The main advance made in plant flux analysis in the last decade is the development of methods to follow the assimilation and subsequent metabolic fate of ^13^CO_2_. While this was proposed as a universal substrate in 2007 [26] it was not until 6 years later that the use of this approach was fully validated at the flux level [27,28]. Early studies were based on the model plant *Arabidopsis thaliana*, partially due to its small size. However, ^13^C data were previously collected though not used for modeling in tobacco [29] and for intact maize plants [30,31]. These experiments are fairly similar in their coverage of central carbon metabolism providing data on the Calvin Benson cycle intermediates, glycolytic intermediates, TCA cycle acids, and amino acids. However, the approach has also recently been adapted in order to determine the rates of protein synthesis [32]. The initial experimental setup was very straightforward consisting of a small sealed system in which a single *Arabidopsis* plant could be grown in air. Importantly, the gaseous environment was set as a flow through system in which the atmosphere surrounding the plant could be rapidly and reproducibly exchanged for a ^13^CO_2_ containing one. Moreover, a mechanism for rapid in-chamber quenching of metabolism was established that allowed near-instantaneous cessation of metabolism via pouring liquid nitrogen over the entire *Arabidopsis* rosette in a manner that did not lead to shading induced changes in the levels of metabolites [27]. In the case of maize a clamp on chamber was placed over a section of the leaf under study and metabolism was quenched by using a metal guillotine [29].

## Compartmentalization

A major challenge in metabolic flux profiling of eukaryotes is the high level of compartmentalization, which implies that a particular label and associated metabolite pool can originate from various, spatially separated organelles or micro-compartments [33,34]. Therefore, for fluxes which take place in multiple compartments, classical approaches which neglect compartmentalization can only determine a global net flux, without resolving the individual, compartmentalized reaction fluxes. As a result, preliminary experiments revealed discrepancies between the measured and anticipated level of labeling of certain metabolite pools. For this reason, in several recent studies the ^13^CO_2_ labeling approach was coupled to non-aqueous fractionation [35,36]. The latter technique is a classical method [37] used in obtaining information concerning the subcellular levels of metabolites – it uses a ‘guilt-by-association’ approach to assign these levels via their co-elution on non-aqueous density centrifugation gradients with markers for the various organelles of the cell. The method was subsequently applied to reveal spatially resolved fluxes in *Arabidopsis* [27].

A different approach is to determine the isotope patterns of macromolecule monomers after incorporation of labeled precursors through proteinaceous amino acids. The approach was applied to resolving fluxes of cell wall and starch synthesis in the cytosol and plastids of soybean seeds [38]. These measurements, together with the higher throughput and accuracy of mass-spectral-based techniques over the ‘classical’ chemical dissection approaches (see e.g. [39–41]), paved the way to the use of the more sophisticated flux modeling approaches that we describe below.

## Overview of established approaches for metabolic flux profiling

We already indicated that metabolic flux profiling integrates data about labeling patterns, monitored via NMR and MS-based technologies, and a model. The model is specified in a form of stoichiometric matrix, *N*, which details the substrates and products of all reactions. The model can further incorporate carbon-transition maps, which capture how the label is (re)distributed from the substrates to the products of a reaction. The consideration of carbon-transition maps partitions a molecular pool into subpools denoting particular labeling patterns (e.g., isotopomers).

**Figure 1:**
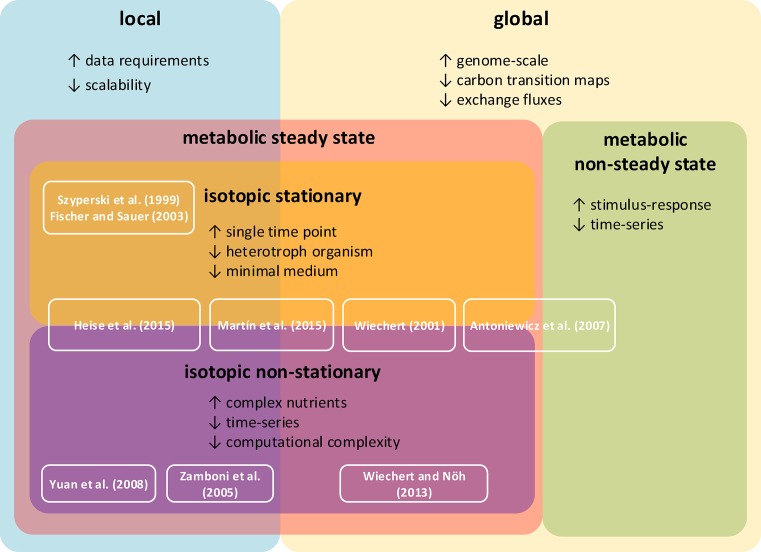
Comparison of flux profiling approaches The modeling approach can be classified into local, which estimates fluxes from proximal labeling patterns, and global, which estimates fluxes in a large-scale network. Experimental setup can be grouped by metabolic steady state and isotopic stationarity. Specific requirements or drawbacks of the different approaches are stated in the corresponding box. Key advantages (↑) and drawbacks (↓) of the different approaches are marked. References to computational methods for flux estimation are shown in white boxes.

The change in the size of a considered pool, *x*, over time can be mathematically described by a system of ordinary differential equations (ODEs), d*x*/d*t* = *Nv*(*x*,*k*), where υ gathers the reaction rates and *k* are parameters on which these rates depend. The existing approaches for metabolic flux profiling can be classified based on several criteria that answer the following questions (see Figure 1): (i) Are global balances of label enforced? (ii) Are the molecular pools in a steady state? (iii) Are the labeling patterns in a steady state?

With respect to the first criterion, the approaches can operate either on a network level, requiring global network balances of label [42–44], or at local level, where only details of individual reactions or reaction pairs are considered [45–47]. Global approaches usually fix the exchange fluxes with the environment [48] or achieve global isotopomer balancing by iterative fitting of extracellular rates [43]. A reduction in computational complexity, especially useful for application of multiple labels, was achieved by decomposition of the network into elementary metabolite units [44]. Following the second criterion, the approaches can be divided into metabolic steady state and non-steady state approaches. In the metabolic steady-state approaches, it must be ensured that there is no change in the pools of the measured components, whereby d*x*/d*t* = *Nv* = 0. By the third criterion, the approaches are grouped into isotopic stationary [47,49], by which there must be no change in labeling patterns, and non-stationary [45,50]. For local isotopic stationary flux profiling, probabilistic equations for branching reactions have been employed in order to estimate ratios of fluxes [47]. Non-stationary flux estimation on a local level can be achieved by modeling the change of unlabeled fractions between substrate and product of a monomolecular reaction using a system of ODEs, referred to as kinetic flux profiling [45]. Extensions of kinetic flux profiling enable flux fitting on a global scale under isotopic stationarity and across multiple compartments [27,51] (Figure 1).

The condition of metabolic steady state is mostly assumed in the prominently applied approaches for metabolic flux profiling. Since the network usually has more reactions than metabolites, the system of linear equations *Nv* = 0 is underdetermined, i.e., when solvable, it has infinitely many solutions. Therefore, data are used to constrain the solution space, resulting in best-fitting fluxes. The global approaches applicable with metabolic and isotopic steady states are computationally the simplest, since they imply solving a system of algebraic equations [46]. There are already several available implementations (including open source) [52]. The data used are obtained from experiments that feed substrates with stable isotopes (e.g., positionally or uniformly labeled glucose, in the case of microorganisms and heterotrophic growth in plants). However, this approach is not applicable with substrates which include a single atom to be labeled, as with ^13^CO_2_, since in this case the isotopic steady state consists only of fully labeled pools, which is not informative. Therefore, it cannot be used to study fluxes in autotrophic growth which is the physiologically relevant scenario for plants (i.e., CO_2_ as a substrate). Since such an approach may also require longer experimental times to achieve isotopic steady state, global isotopic non-stationary approaches at metabolic steady state have been developed and refined [42].

The data employed in the latter are obtained from experiments in which the fed substrate is switched from unlabeled to labeled, and the time evolution is monitored in short time intervals. These approaches are particularly relevant to resolve fluxes in linear pathways and cycles, which are typically arising in secondary metabolism. The main challenge is ensuring that the system is at a metabolic steady state, particularly if the interaction between primary and secondary metabolism is to be investigated. These approaches simulate the time evolution of label incorporation and aim at finding fluxes which best fit the experimentally obtained time-series of the labeling patterns. Therefore, the task of fitting a system of ODEs whose size does not scale with the number of reactions and metabolites considered quickly becomes daunting. Despite these challenges, there are software solutions which can be used with systems of sizes encountered in present experiments [53,54]. Local non-stationary approaches are developed to address the numerical and fitting issues arising in global non-stationary approaches at metabolic steady state. They are used to estimate fluxes for individual reactions by employing data about the time-series of the non-labeled metabolic pools [45], or use time-series of all labeling patterns to estimate flux ratios [46]. The former approach is extended to estimate canonical fluxes in a photosynthesizing *A. thaliana* rosette [27].

Since metabolic stationarity may not be easy to maintain, particularly in cases where response to stimuli is investigated, approaches based on metabolic non-steady states have also been devised, although their application remains confined to a few case studies [44]. This succinct overview of approaches based on integration of labeling patterns and a model highlights the developments in the field toward estimation of a small subset of fluxes (or their relations), rather than the entire distribution of fluxes in a network. The question is then what these fluxes may tell us about the activities of other reactions? To this end, fluxomics data have been successfully used in the constraint-based modeling framework [55], providing one possibility to determine fluxes at a genome-scale level, albeit at the cost of introducing additional assumptions. Moreover, the uncertainties associated with estimated fluxes are often large [55], which can be addressed by integrating complementary sets of omics data within constraint-based approaches. In the following, we first review how uncertainties in flux profiling can be assessed, and then briefly review the developments of constraint-based approaches and the possibilities they offer for flux profiling in different plant organs, tissues, and cell types.

## Error estimation and uncertainties in flux profiling

The large uncertainties associated with estimated fluxes are a major challenge of flux profiling approaches. Fluxes of individual reactions can rarely be pinpointed to a single value, and consequently, multiple alternative flux distributions are in agreement with the measured isotope patterns. One possibility to reduce the uncertainties is to integrate transcriptomics or proteomics data within the constraint-based framework using supervised or non-supervised approaches (reviewed in [56] and extended in [57,58]). Depending on the experimental setup, unlabeled metabolomics data can also be integrated with constraint-based models, which can lead to a substantial reduction of the uncertainties associated with estimated fluxes, discussed in the section ‘Integration of unlabeled metabolomics data’.

It was also pointed out that the use of different commonly used statistics for calculating the confidence intervals of estimated fluxes can yield different results, particularly when the relationships between measurements and fluxes are nonlinear [59]. The authors demonstrate that these differences lead to different error estimations in practice. Further, they propose an alternative approach for error estimation, by first calculating the posterior probability distribution of the measurements using Markov Chain Monte Carlo sampling, and then using Bayesian statistics to calculate credibility intervals. This approach has the advantage that its error does not depend on approximation assumptions, since the credibility intervals directly correspond to the width of the posterior probability distribution. Moreover, the numerical precision of intervals can be increased arbitrarily by investing more computational power. The authors use a labeling experiment with a model of *Escherichia coli* central metabolism to show that the posterior probability distributions for some reactions indeed differ significantly from the Maximum Likelihood Estimator distributions obtained by parametric bootstrapping [60]. However, although its computational complexity seems to be improved in comparison with parametric bootstrapping, it is still significantly less efficient compared with methods based on fitting of fluxes, i.e., Fisherian statistics [61] and profile likelihoods [62].

## Highlighted advances of metabolic flux profiling at a genome-scale level

Using genome sequencing information from a variety of organisms, an increasing number of genome-scale metabolic networks have been reconstructed [63]. The basic approach aims at establishing a comprehensive collection of all enzyme-catalyzed and transport reactions taking place in a cell, mainly from the biochemical literature and by transfer of reactions using sequence homology of genes in related organisms [64]. While such reconstructions provide a vast collection of the available knowledge on the metabolism of an organism, they have also enabled computational studies for generating and testing novel hypotheses relating genotype and reaction fluxes (reviewed in [65]). Compared with the use of smaller models, as in the classical approaches of metabolic flux profiling reviewed above, such large-scale approaches have the advantage of providing a systems view on metabolism. For example, genome-scale approaches implicitly take into account the possibly unexpected interactions between seemingly unrelated pathways, such as through the broad use of cofactors. The approaches rely on a given metabolic network structure, *N*, which contains the stoichiometric coefficients of all considered exchange, transport, and enzymatic reactions. By imposing *Nv* = 0, only those reaction rates υ which guarantee metabolic steady state are considered. A specific flux distribution υ satisfying *Nv* = 0 then corresponds to a unique metabolic steady state of the system.

The now classical way of addressing the problem of an infinite number of possible flux distributions satisfying *Nv* = 0 is given by flux balance analysis (FBA, [66])—the essence of constraint-based modeling. FBA assumes a cellular objective, such as maximization of growth or ATP production, and only considers flux distributions which optimize this objective. While this constraint-based approach was largely successful in modeling microbial systems (see e.g. [67,68]) and several competing objectives of bacteria have been identified [70], it is unlikely that metabolism of eukaryotes, like plants, is optimized for one specific objective [69,71]. Moreover, even when using an objective function, still many alternative flux distributions satisfy the objective [72,58]. Therefore, numerous attempts have been made to integrate additional levels of information with metabolic networks, such as thermodynamics [73], gene regulation [74], context-specific gene expression [75], and data obtained from various omics technologies [57,76]. Moreover, integrating data sets from a given experiment can be used to obtain a context-specific model variant, which may significantly differ from the generic genome-scale model which considers all reactions, independently of their observed activity in a given context [56].

Since the fluxes, υ, of a metabolic network are directly modeled, fluxomics data provide the most direct way of complementing and validating constraint-based approaches. The simplest way of integrating estimated fluxes from measurements is to limit the allowable fluxes in υ to the flux values obtained from fitting labeling patterns, ideally also allowing for measurement errors. This approach has been frequently applied in modeling of genome-scale metabolic networks and was used for validating the predictions, refining a metabolic model, or for narrowing down the range of possible flux distributions [55,77,78,79]. For example, Segrè et al. [80] integrated data from fluxomics experiments of *E. coli* central metabolism [81] with an early genome-scale metabolic network [82] to validate their computational approach for prediction of knockout phenotypes, called Minimization of Metabolic Adjustment. They found a good agreement between FBA-based flux predictions on the genome-scale and those estimated using flux analysis in wild-type *E. coli* under one nitrogen and two glucose limited conditions. Moreover, they were also able to find a better agreement of the predictions using the proposed approach in two knockout mutants, as compared with the naïve approach based only on FBA. The same technique of integrating ^13^C fluxomics measurements was used to study the role of gene duplication using a genome-scale metabolic network of *Saccharomyces cerevisiae* [83].

In a slightly different approach, intracellular flux measurements from Fong et al. [84] were used to identify model errors which could explain the observed discrepancies between simulations and the outcome of adaptive evolution experiments for engineering lactate production using *E. coli* [85]. This question is relevant since the fluxes estimated based on fitting the labeling patterns depend on the model used, and iterative model selection strategies are not currently employed. The study identified multiple sets of one to four reactions whose removal from the model greatly improved the predictions on growth rate and intracellular fluxes in the producing strains. The removal of the identified model reactions was further justified by the observed significant down-regulation of the corresponding enzyme-coding genes in the evolved strains.

However, these simple approaches of integrating estimated fluxes with genome-scale models have the drawback of combining two disparate modeling strategies: first, the estimation of fluxes from labeling patterns with a simplified model using flux profiling approaches, described above, and subsequently their direct integration with a genome-scale model. The use of two different models may lead to estimation of fluxes which, on a genome-scale, do not correspond to a best fit to the labeling data, or are inconsistent with the modeling assumptions. For example, it was found that the fluxes estimated using flux profiling in a small model were within the flux ranges predicted by FBA in a genome-scale model. However, the individual flux distributions ultimately obtained from sampling of the genome-scale flux distributions were substantially different from those estimated using the small model [79]. This indicates that caution is necessary when integrating fluxomics data with genome-scale networks.

To remedy these limitations, a few computational approaches have been developed which directly integrate the labeling patterns or isotope pool sizes with genome-scale metabolic networks. For example, by adding artificial ‘dummy’ metabolites which connect two reactions sharing a common product, Choi et al. [86] were able to represent the fractional contribution of isotopomers within the constraint-based modeling framework. This approach allows for directly constraining genome-scale flux distributions by the relative fluxes of converging pathways from measured isotopomer distributions. The authors demonstrated that inclusion of labeling data from Fischer et al. [87] with a genome-scale model of *E. coli* leads to improved flux predictions for some reactions in central metabolism, although most predicted fluxes are similar when not using the labeling data. However, when the approach was applied to a small-scale model of *E. coli* central metabolism, predicted fluxes were noticeably more accurate compared with flux predictions with a genome-scale model using the same data set. The approach has so far been validated almost exclusively with the small-scale model [86]. Hence, it remains to be demonstrated whether the approach provides reliable flux estimations on a genome-scale.

A novel method for directly integrating ^13^C fluxomics data with genome-scale metabolic networks was developed by Martín et al. [88]. The approach, called two-scale ^13^C metabolic flux analysis (2S-13C-MFA), relies on the assumption that carbon flows from core to peripheral metabolism, which is supported by existing successful applications of flux analysis in central metabolism, since it relies on a similar assumption. Importantly, the approach does not require a cellular objective function, such as maximization of growth, and should hence be applicable to higher organisms. The approach relies on two different modeling strategies for core metabolism, which is modeled by fitting metabolite distribution vectors obtained from labeling data, and peripheral metabolism, which is modeled based only on the steady-state principle while enforcing minimal carbon flow into the core. In an iterative approach, the goodness of fit to the labeling data is evaluated for core fluxes, and reactions from outside the core are included based on their contribution to the error of the fit. The approach circumvents the need for selecting which reactions may affect observed labeling patterns and would consequently need to be included in the model, as required for classical ^13^C metabolic flux analysis. Moreover, since carbon flows primarily from central to secondary metabolism, peripheral reactions are likely to be strongly constrained using the two-scale approach. Importantly, global ATP and cofactor balances are implicitly taken into account by the approach. This is especially relevant in metabolically engineered strains, where fluxes in secondary metabolism can dramatically affect these balances. By using ^13^C flux data for 94 reactions in *E. coli* central metabolism, the authors show that reactions which are not commonly included in models used for flux profiling are implicitly modeled as part of core metabolism by the approach, which leads to a dramatic improvement of the fit to within the range of experimental error.

## Integration of unlabeled metabolomics data

Two sets of approaches have been recently proposed to allow the integration of unlabeled metabolomics data for the purpose of flux estimation on a genome-scale. The first, termed TREM-Flux, integrates partial time-resolved metabolomics and transcriptomics data with the constraint-based approach to predict non-steady-state fluxes in response to a perturbation, such as a treatment or a change of environmental condition, without the need of kinetic parameters [77]. The authors apply the approach to *C. reinhardtii* treated with rapamycin under mixotrophic and autotrophic conditions. They show that the measurement of 45 of 1063 metabolites in the genome-scale model is sufficient to dramatically narrow down the space of possible flux distributions and predict phenotypes that are consistent with differences in metabolites across treatment and environmental conditions. The approach was recently extended to likely reduce noise in the consecutive time points and produce sparser solutions [89].

Following a similar line of thought, a method called iReMet-Flux allows to predict fluxes from relative metabolomics data under two steady-state conditions [90]. The approach uses the assumption of mass action kinetic to remove the dependence of flux ratios between conditions on the kinetic rate constants. The flux ratios derived from relative metabolite measurements are then integrated with the constraint-based approach, while unconstrained fluxes are assumed to be similar under the considered conditions (however, without requiring a reference flux distribution, as with the Minimization of Metabolic Adjustment [80]). The authors also provide an approach to derive flux ranges by allowing for uncertainties in the measured parameters. The approach was applied to photo-autotrophically grown *A. thaliana* and four photorespiratory mutants undergoing high-to-low CO_2_ acclimation, demonstrating that the predicted flux differences are in line with observed differences in phenotypes of the mutants. It also allowed making statements about differentially behaving fluxes as the main aim of large-scale profiling technologies – the comparison of biological scenarios to determine the mechanisms employed by a system to respond to perturbations. However, such an approach comes at the cost of assuming a particular form of reaction rates (i.e., mass action), although it allows for the consideration of changes of enzyme levels.

## Discussion

Despite recent advances in approaches which allow for increasing the percentage of model reactions whose fluxes can be estimated by integration of heterogeneous data, there are remaining challenges in flux profiling on a genome-scale level. We believe that the combination of local approaches for flux estimation from labeling data in combination with the constraint-based modeling framework therefore provides one possibility to address the problem of flux profiling at a genome-scale. To facilitate research in this direction, atom transitions on a genome-scale need to be integrated into the existing curated models. In addition, such an approach will render it possible to do flux estimation for a wider range of experimental setups without the need of a particular cellular objective to be optimized (e.g., biomass yield). For instance, a recent simplified model of metabolism of *E. coli* considers atom maps for secondary metabolism [55]; although the precision of flux estimates does not suffer, the model simplification relies on optimizing an objective (per parsimonious FBA).

There are at least two avenues which can be followed to reduce the flux space by combining flux estimates from local approaches with constraint-based approach: (1) develop approaches which consider the total pool sizes of metabolites to obtain bounds on fluxes, (2) combine data on reaction rate constants with data on enzyme abundances to limit fluxes. Like iReMet-Flux, the first opportunity necessitates making assumptions about the flux form and its dependence on metabolite pool sizes [91]. Thereby, it can be used to reduce the degrees of freedom in flux estimation, provided metabolomics data of sufficient coverage are available. However, this approach requires a considerable amount of data to develop the models for flux in terms of metabolite concentrations. In the second avenue, the flux estimates obtained from the local approach can be combined with approaches in which additional bounds on fluxes, in terms of rate constants and protein abundances (as in [92]), are employed to reduce the flux space.

Despite these possibilities, combining the flux estimates to increase the coverage of flux predictions faces the issue of integrating predictions from heterogeneous models and computational approaches, with their specific set of assumptions.

For example, 2S-13C-MFA has so far only been applied to microorganisms [88,93,94], and it cannot be directly applied to the use of other than carbon labeled substrates. TREM-Flux requires time-resolved experimental data, while iReMet-Flux relies on comparison of two different metabolic states under the assumption of mass action kinetic.

We envision that future computational approaches will be developed which maximize the use of information obtained from directly using isotope labeling patterns by integrating atom transition maps with genome-scale metabolic networks, while limiting the assumptions made to essential biochemical and thermodynamic constraints. Such an approach, closest to the metabolomics data generated from different studies, rather than the flux estimates obtained by using a particular combination of model and computational approach, will lead to increased understanding of genotype–phenotype relationships and the molecular mechanisms underlying complex metabolisms in the near future.

## Abbreviations

FBA: flux balance analysis
MS: mass spectrometry
NMR: nuclear magnetic resonance
ODE: ordinary differential equation
